# Is gold a safe haven for the dynamic risk of foreign exchange?

**DOI:** 10.1186/s43093-021-00101-9

**Published:** 2021-11-01

**Authors:** Kuan-Min Wang, Thanh-Binh Nguyen Thi, Yuan-Ming Lee

**Affiliations:** 1 Department of Finance, Overseas Chinese University, 100 Chiao Kwang Road, Taichung, 40721 Taiwan; 2 Department of Accounting, Chaoyang University of Technology, 168 Jifong E. Road, Wufong Township, Taichung County, 41349 Taiwan; 3 Department of Finance, Southern Taiwan University of Science and Technology, No.1, Nantai St, Yung-Kang City, Tainan, Taiwan

**Keywords:** Gold price, Foreign exchange risk hedge, Time-varying parameter, Panel VECM model, Impulse response, C32, E44, G15

## Abstract

This paper uses the panel data of 15 countries from 2009 to 2020 to construct the time-varying parameter panel vector error correction model for testing the hypothesis of dynamic hedging characteristics of gold on exchange rate. As the existing literature has never considered that the foreign exchange risk hedged by gold is dynamic, this study can fill the research gap in this area. The empirical results show that: First, gold can partly hedge against the depreciation of the currency in the long run; second, gold is unable to hedge against the risk of the exchange rate when considering dynamic hedging effects in the short run; third, when facing unexpected shocks, the impulse response shows that the gold returns have reversible reactions compared to exchange rate fluctuations; therefore, gold can regard as a safe haven for foreign exchange markets; Finally, the government, as well as investors should always be concerned about these dynamic risks and formulate effective hedging strategies to control the currency uncertainty.

## Introduction

It is critical for multinational companies, export–import businesses, and investors making foreign investments to understand the impact of exchange rate risk on their profitability of trade and investment deals. How to mitigate the exposure risk of the exchange rates is an issue that is often discussed or tried to solve empirically and theoretically by economists. Due to the uncertainty of domestic and international stock markets and the fact that the bond yields of developed countries are relatively lower than those of developing countries, investors continue to increase their investment in new assets of emerging markets. They have to deal with the risks related to international investment and domestic assets. The risks of domestic inflation and capital flow are relatively substantial when facing exchange rate fluctuations. To effectively manage the dynamic risk of foreign exchange rates, investors and governments must exercise centralized oversight over their foreign exchange hedging strategies where gold is essential. It acts positively in times of uncertainty and market volatility.

Bordo and Rockoff [7] argued that the gold standard represented a "good housekeeping seal of approval" from 1870 to 1914. The USA effectively folds up the Bretton Woods system and renders the dollar a freely floating fiat currency when terminating the convertibility of the U.S. dollar to gold in 1971. However, several studies in the literature have since provided the potential of gold to serve as a hedge or safe-haven asset against exchange rate risk (such as Beckers and Soenen [6]; Sjaastad and Scacciavillani [50]; Sjasstad [49]; Capie et al. [13]; Pukthuanthong and Roll [39]; Joy [23]; Wang and Lee [52]; Zagaglia and Marzo [56]; Iqbal [22]; Sakemoto [43]; Qureshi et al. [40]; Aftab et al. [1] and Ranaldo and Söderlind [41]). Several studies have examined the safe-haven feature of gold by exploring if gold help withstands the price volatility of assets that are more vulnerable to market shocks in short-term (such as Akbar et al. [2]; Baur and Lucey[5]; Bouri et al. [8]; Reboredo [42]; Shahzad et al. [45]; and Salisu et al. [44]).

This study aims to find a solution for dynamic risk management for policymakers, corporate managers, and investors. For many years, gold has been regarded as a tradable financial asset and a safe haven from market turmoil. As gold added to the asset portfolio can reduce the volatility of returns and avoid the exchange rate's fluctuation risk, maintaining a positive return on the portfolio is one of the main reasons why gold attracts investors and managers. At the same time, changes in market information continuously occur. According to the principle of the asset portfolio, assets with low or negative correlations are select to form a portfolio in the hope of reducing risks and maintaining returns. Investors put a portion of their portfolios in gold mainly because other financial assets, such as stocks and bonds, cannot hedge against the depreciation of their currencies. Hence, if gold can hedge against exchange rate risk, putting gold in the portfolio will make it more efficient.

Moreover, economies engaged in global trade and international finance might be influenced by their gold holding levels. Governments hold gold in their reserves for three primary reasons; risk mitigation, inflation hedge, and facilitation on economic stability and growth. National banks of many countries seek to purchase gold for hedging against a weakening dollar or any other fiat currency. In other countries where their domestic currencies are weak, central banks add gold to their holdings for seeking the hedge by purchasing dollar-denominated gold.

The ability to hedge against the exchange rate risk of gold is based on two perspectives. First, gold can resist the change in the purchasing power of a country's currency. When the price index rises, the gold price expressed in its currency increases simultaneously and at the same level, which is called the perfect internal hedge. If the increased level in gold price is less than the increase in the price index, it is the partial internal hedge. Second, the rise of gold price in the domestic currency is equal to the decrease in the rate of the domestic currency to the foreign currency per unit, which is regarded as the perfect internal hedge, conversely in the same way, if the increase is less than the depreciation level, it is a partial external hedge. The main focus of this study is the first prospective, and it further examines whether gold is a safe haven for foreign exchange markets and if gold can evade the impact of foreign exchange market shocks.

The best way is to examine the relationship between gold and other assets and realize gold investment performance from a global perspective. So far, however, the existing empirical literature has not considered that the correlation or risk factor changes dynamically. Also, it may not be appropriate to discuss gold hedge against the exchange rate for a single country as the risk factor under international risk sharing and financial integration may change with time and state. How to propose relative risk diversification strategies and measures at the right time and condition for the dynamic relationship is very important in practical operations.

Therefore, this study uses a multinational model based on the time-varying TVP-PVECM that can explore the hedging characteristics of gold on the dynamic risk of the exchange rate and further analyze the risk hedging performance and impulse response. This study first tests whether gold can hedge the devaluation of a country currency in the long run; then, examine whether gold can hedge against the risk of a country currency depreciation in the short run; finally, explore whether gold is a safe haven for the foreign exchange market.

Three main empirical findings emerge. First, significant evidence shows the power of gold in hedging currency depreciation in the long run. Second, the hedge of gold against the short-term risk of the exchange rate is not supported by estimating the linear model. Third, the effect of the short-term impulse response is more significant than the long-term, and gold is the safe haven of the foreign exchange risk. This study contributes to the literature in several ways. In terms of research topic, it designs multinational research and involves the impact of cross-national contamination effects. In terms of research methodology, compared to the traditional fixed-parameter model, the estimation of the variable parameters can better grasp the influence of dynamic time and state and provide investors with a reliable gold diversification mechanism on exchange rate risk in practical operation. Besides, to analyze the response of variables to the other shocks with different lengths of time and intensity, we perform impact response analysis. The impulse response analysis is a crucial tool for testing the dynamic effects between variables in VAR models. As traditional model parameters do not change over time, we develop the TVP-PVECM to test the dynamic interaction between variables in another dimension. The impact response effect at all-time points can compute using the estimated time change parameters.

Our empirical findings reveal the safe haven of gold on exchange rate risk and focus on its dynamic hedging effectiveness compared to previous research. The remainder of the paper is organized as follows. In "Review of literature" section, we review the literature on the gold hedge. In "Methods" section, we develop the theoretical model and research methodology. The empirical results are reported in "Results and discussion" section. "Conclusion" section is the conclusion.

## Review of literature

Discussing the interactions between the gold price and macroeconomic variables (such as exchange rate, interest rate, income), Ariovich [3], Fortune [19], Dooley et al. [18], Sherman [46–48], Sjaastad and Scacciallani [50] and Sjaastad [49] use the time series study, most of which are performed with linear models. The disadvantage of using a linear model is that it is impossible to predict the relationship between variables in different situations, such as exchange rate fluctuations. Kyrtsou and Labys [26] suggest that if a bidirectional nonlinear relationship between variables is determined, a linear model used for estimation may result in errors. Generally, volatility clustering always exists in financial data, and there may be beaten and cyclic nonlinear phenomena at certain times. The empirical estimation may produce bias without consideration of this phenomenon. Thus, Baker and Van Tassel [4], Diba and Grossman [17], Koutsoyiannis [23], and Pindyck [37] use dynamic models to discuss factors affecting the volatility of the gold price. Besides, Chappell and Dowd [14], Kolluri [24], Laurent [27], Mahdavi and Zhou [31], and Moore [33] explore the long-term and short-term relationship between gold prices and commodity price indices and discuss the effectiveness of gold on hedging inflation risks.

However, studies relevant to gold nexus dynamic exchange rate risk are few. Joy [23] found that gold is an effective hedge against currency risk associated with the U.S. dollar. Wang et al. [55] performed an empirical study on Japan. They found that the asymmetry caused by exchange rate fluctuations affects the effectiveness of using gold to avoid the yen devaluation. Wang and Lee [52] used a nonlinear model to test the inflationary hedge effectiveness of gold in Japan and the USA; they found that the price rigidity of transaction costs would affect the anti-inflation effect of gold. Because the above literature uses time series models for analysis or only for a single country, there are limitations in empirical explanation. Therefore, Wang [51] and Wang and Lee [53] use a multinational panel asymmetry model to conduct cross-national analysis and characterize the gold hedge. Wang and Lee [53] further found that the hedging effectiveness of gold on exchange rate risks is different due to the asymmetric information caused by exchange rates in gold major producing countries and major demanding countries. Qureshi et al. [40] use wavelets at multiple time horizons and find that gold acts as a consistent short-run hedge against exchange rate hence validating the exchange rate destruction hypothesis. Their finding suggests that the central banks also need to keep other safe-haven assets in reserves as the hedging ability of gold is only limited in the short run. Besides, the role of gold in providing protection against currency risks is also confirmed with quantile regression. Their results also suggest that gold has a lead effect on the exchange rate, However, this effect switches over different regimes. Aftab et al. [1] who use the dynamic conditional correlation-multivariate generalized autoregressive conditional heteroscedasticity model to test the gold link with equity and currency market, suggest that gold acts as a hedge and safe haven against Asian currencies except for China and Hong Kong, and thus, it still preserves its monetary role.

The seminal article of Ranaldo and Soederlind [41] uses the factor model to capture linear and nonlinear linkages between currencies, stock and bond markets as well as proxies for market volatility and liquidity. They find that the Swiss franc and Japanese yen appreciate against the US dollar when US stock prices decrease and US bond prices and FX volatility increase. These safe-haven properties materialize over different time granularities (from a few hours to several days) and nonlinearly with the volatility factor and during crises. Baur and Lucey [5] study the constant and time-varying relations among US, UK, and German stock, bond, and gold returns to investigate whether gold acts as a hedge and a safe haven. They find that gold is a hedge against stocks on average and a safe haven in extreme stock market conditions. A portfolio analysis further shows that the safe-haven property is short-lived. Reboredo [42] uses the copulas approach to assesses the role of gold as a hedge or safe haven against oil price movements. Empirical results reveal that gold cannot hedge against oil price movements, but can act as an effective safe haven against extreme oil price movements.

Recent research on the gold issue of Omane-Adjepong and Boako [35] examines long-range dependence in global gold returns and volatilities and applies a semi-parametric test. The study that accounts for the effects of structural breaks and economic shocks finds no significant evidence of long-range dependence in returns (volatilities) for the full-sample gold data. However, Mo et al. [32] examine the dynamic linkages among the gold market, U.S. dollar, and crude oil market. The analysis also delves more deeply into the effect of the global financial crisis on the short-term relationship. The DCC-MGARCH model is employed to investigate the time-varying long-term linkages among these markets. The Krystou–Labys nonlinear asymmetric Granger causality method is used to examine the effect of the financial crisis. This study found that (1) there is a long-term dependence among these markets; (2) the dynamic gold–oil relationship is always positive and the oil–dollar relationship is always negative; and (3) after the crisis, there exist a positive nonlinear causal relationship from gold to U.S. dollar and U.S. dollar to crude oil, and a negative nonlinear causal relationship from U.S. dollar to gold.

In this study, we extend the research method of gold-related literature by Wang and Lee [52], Wang et al. [55], Wang [51], Wang and Lee [53], and Wang et al. [54]. In terms of empirical research, we can compare the magnitude of the bias response and consider the dynamic process of the time-varying fluctuation and correlation. To simulate the effects of financial crisis shocks, we fixed an original shock size equal to the average of the random changes of time series during the sample period while using the synchronization correlation at each time point.

To estimate the innovation process of deferred variable regression, we use the current to future period to compute the time-varying coefficient. The time level and time point are selected in the final period of the sample, and a multi-dimensional impulse response graph is generated. Minimal effort has been made in the prior literature to use multinational panel data in which a specific country at a particular time is selected for impulse response analysis. In the empirical process, we can verify the impulse response of exchange rate fluctuations to the gold price interference at different frequencies in the future, compare the effects of short-term and long-term impulse responses, and provide pieces of evidence to the existing literature.

## Methods

### Theoretical model

We build a model based on Wang and Lee [53], which is an extension of Clements and Fry [15], Wang and Lee [52], and Wang [51]. First, purchasing power parity (PPP) theory constructs the relationship between the gold price and the exchange rate. Suppose purchasing power parity holds, a generalized global model of gold equilibrium price can be constructed. Assuming that the relative PPP theory is established by arbitrage in the international financial market, then the gold price model can be set up as follows:

1 $$Q^{s} = Q^{s} (G_{d} /P_{d} ),\quad Q^{d} = Q^{d} (G_{f} /P_{f} ),\quad Q^{s} = Q^{d} ,\quad G = EG^{*} {(1} + X{)}$$

where $Q^{s}$ is the gold supply, $G_{d}$ is the gold price index presented in the local currency,$P_{d}$ is the domestic price index, $Q^{d}$ is the domestic consumption of gold, $G_{f} /P_{f}$ represents the gold price faced by foreign consumers, $E$ is the spot exchange rate of the domestic currency to one unit of foreign currency. $X$ is price spread between domestic prices and foreign prices. Suppose this price spread is fixed and Eq. (1) is differentiated, let (^) be the percentage (%) chance of the variable (such as $\hat{Z} = \Delta Z/Z$). Basing on these settings, Eq. (2) can be derived as follows:

$$\hat{G}_{f} /P_{f} = \theta \hat{R}$$

$\theta = \phi /(\phi - \vartheta )$ indicates the ratio of supply elasticity to excess supply elasticity, $\phi$ is the price elasticity of supply and $\vartheta$ is the price elasticity of demand. $R = P_{d} /EP_{f}$ represents the real exchange rate. If the supply elasticity $\phi \ge 0$, the demand elasticity $\vartheta \le 0$, and $0 \le \theta \le 1$, an increase in *R* will indicate the real appreciation of the local currency (gold-producing country). Equation (2) is the price rule of gold, which means that the rate of change in the relative international price of gold is the ratio $\theta$ of the change in the real value of the domestic currency.

Next, we use PPP to link the domestic and foreign prices of gold; if $X$ = 0,$G = EG^{*}$. By dividing both sides by $P_{{\text{s}}}$ and using the relationship of $R = P_{{\text{d}}} /EP_{{\text{f}}}$, the equation $G_{{\text{f}}} /P_{{\text{f}}} = R(G_{{\text{d}}} /P_{{\text{d}}} )$ can be further derived. This equation provides a connection channel between the relative prices of the domestic and foreign countries, which can also be regarded as a substantial PPP. In order to settle the market, the trade deficit and excess supply must offset international prices to stimulate demand. When market-clearing occurs $\phi (\hat{G}_{d} /\hat{P}_{d} ) = \vartheta (\hat{G}_{f} /\hat{P}_{f} )$, Eq. (3) is derived in the following form:

3 $$\hat{G}_{f} /P_{f} = (\phi /\vartheta )(\hat{G}_{d} /G_{d} ) = - \left[ {\theta /\left( {1 - \theta } \right)} \right](\hat{G}_{d} /G_{d} )$$

Then, the real PPP reflected in the relationship between domestic and foreign gold prices is $G_{f} /P_{f} = R(G_{d} /P_{d} )$. In the general equilibrium framework, Eq. (4) can be derived from Eq. (2), (3), and $R = (P_{d} /EP_{f} )$:

4 $$\hat{G}_{d} /G_{d} = - \left[ {(1 - \theta )/\theta } \right](\hat{G}_{d} /P_{f} ) = - (1 - \theta )\hat{R}_{d} = (1 - \theta )(\hat{E} + \hat{P}_{f} - \hat{P}_{d} )$$

All other terms and conditions remain unchanged, the relationship between gold price changes and exchange rate fluctuations is:

5 $$\hat{G}_{d} = f(\hat{E})$$

We incorporate the elasticity of demand and supply into Eq. (4), and the exchange rate hedging equation of gold in a country is as follows:

6 $$\hat{G}_{d} /G_{d} = (1 - \theta )\hat{E} = - [\vartheta /(\phi - \vartheta )]\hat{E}$$

Suppose the domestic and foreign price indices remain unchanged. The price change is 0 ($\hat{P}_{f} = \hat{P}_{d} = 0$), then $\hat{G}_{d} /G_{d} = (1 - \theta )\hat{E} = - [\vartheta /(\phi - \vartheta )]\hat{E}$; the coefficient of exchange rate hedge of gold is $- [\vartheta /(\phi - \vartheta )]$, which indicates that the demand elasticity and supply elasticity of gold affect hedging effectiveness. Suppose in Eq. (6), the hedging coefficient of gold is characterized with varying hedge effectiveness and impact relationship when the time changes. Based on the above inferences, we argue that the elasticity of demand and supply will change over time, then cause different hedging characteristics. For empirical analysis, we set Eq. (5) as below:

7 $$\hat{G}_{it} = f(\hat{E}_{it} )$$

where $\hat{G}_{it}$ is the logarithmic return of the gold price expressed in the domestic currency of a country $\hat{E}_{it}$ is the volatility of the exchange rate (defined as the U.S. dollar per domestic currency). If $f^{\prime}$ > 0, gold returns can hedge the risk of losing the purchasing power of a country's currency. The positive $\hat{E}_{it}$ implies a country's currency depreciation or an increase of the gold price in domestic currency. If the level of growth is less than the one of depreciation, it is an incomplete or partial hedge. Otherwise, if the level of increase is greater than the depreciation, it is a complete hedge.

When a country's monetary authority intervenes from time to time, there may be information asymmetry and additional transaction costs in the market. At this time, the demand and supply elasticity will be affected, and these effects vary over time, causing the different impact of the hedging effect.

## Empirical model

This study uses the dynamic panel VAR model developed by Canova et al. [12], Canova and Ciccarelli [10], and Canova and Ciccarelli [11] for analysis. The multinational TVP-panel VAR. is as follows:

8 $$y_{it} = {\varvec {D}}_{it} (L)Y_{t - 1} + e_{it} ,$$

where $i = 1, \ldots ,N$ is the country number, $t = 1, \ldots ,T$ present time period, $L$ is lag operator, ${\varvec {D}}_{it} (L)$ is the $p$-order delay polynomial. $y_{it}$ is for any country i that includes $G$ ($G \times 1$ vectors) endogenous variables. $Y_{t - 1}$ contains all countries’ ($G \times 1$ vectors) endogenous variables for the model and error term ($G \times 1$ vectors)$e_{it}$. At each point of time, each equation of the panel VAR includes $k = NGp$ coefficients. For each country, the lag polynomial can be transposed into a coefficient matrix of $G \times k$ vectors. The total coefficients of the entire system $NGk$ must be estimated at each time point of Eq. (9). This shows that we must have a reasonable structure for a large dimensional model to ensure the feasibility of the estimation. Assuming that the system setting in Eq. (2) is:

9 $$Y_{t} = \sum\limits_{j = 1}^{p} {D_{jt} Y_{t - j} + E_{t} }$$

where $D_{jt}$ is $NG \times NG$ coefficient matrix that includes the lag polynomial. We simultaneously define $Y_{t} \equiv \left( {y^{\prime}_{1t} , \cdots ,y^{\prime}_{Nt} } \right)$ and $E_{t} \equiv \left( {e^{\prime}_{1t} , \cdots ,e^{\prime}_{Nt} } \right)^{\prime }$, where $E_{t} \sim \left( {0,\Omega } \right)$. Then the model can be combined as:

10 $$Y_{t} = D^{\prime}_{t} X_{t} + E_{t}$$

In which $D^{\prime}_{t} \equiv \left( {D_{1t} ,D_{2t} , \cdots ,D_{pt} } \right)$ and $X^{\prime}_{t} \equiv \left( {Y^{\prime}_{t - 1} ,Y^{\prime}_{t - 2} , \cdots ,Y^{\prime}_{t - p} } \right)$. Vectorizing the two sides of Eq. (10) can lead to seemingly unrelated regressions such as (11):

11 $$Y_{t} = Z_{t} \delta_{t} + E_{t}$$

where $Z_{t} \equiv {I}_{NG} \otimes X^{\prime}_{t} ,\delta_{t} \equiv {vec}\left( {D_{t} } \right) = \left( {\delta^{\prime}_{1t} , \cdots ,\delta^{\prime}_{Nt} } \right)^{\prime }$ and ${I}_{NG}$ is the $NG$ identity matrix. As mentioned above, estimating Eq. (11) would be difficult to estimate without further restrictions, so we use the method of Canova and Ciccarelli [10] to impose some restrictions on parameter $\delta_{t}$. It is assumed that there are $r < < NGk$ unobservable ergodic states collected in $\left\{ {\theta_{t} } \right\}_{t = 1}^{T}$ to obtain the characteristics of the coefficients that vary with time:

12 $$\delta_{t} = \Xi \theta_{t} + u_{t} ,$$

where $\Xi$ is a $NGk \times r$ matrix and $u_{t}$ is the error term of the decomposition $\delta_{t}$, where $u_{t} \sim {N}\left( {0,\Sigma \otimes V} \right)$. Limits are included in the matrix $\Xi$ to ensure the feasibility of the estimate. To obtain the interdependence of dynamic and transnational cross-sectional data at the same time point, we use a similar approach to Canova and Ciccarelli [11], which assumes that there is a global common component, national relevant component, and variable relevant component, such as $\Xi \theta_{t} = \Xi_{g} \theta_{g,t} + \Xi_{c} \theta_{c,t} + \Xi_{\upsilon } \theta_{\upsilon ,t}$, where $\theta_{g,t}$, $\theta_{c,t}$, and $\theta_{\upsilon ,t}$ are the global common component, national relevant component, and variable relevant component that change over time, respectively. Relative to the unobserved coefficients of panel VAR $\Xi_{g}$, $\Xi_{c}$, and $\Xi_{\upsilon }$, which are matrixes $NGk \times 1$, $NGk \times N$, and $NGk \times G$, respectively. To replace each of the coefficients in the modeling _*NGk*_ process, we estimate $r = 1 + N + G$ in $\left\{ {\theta_{t} } \right\}_{t = 1}^{T}$. This design allows us to distinguish between heterogeneity across countries, changes in the relationship between variables, and the globalization factor that affects all country variables. We substitute Eq. (12) into (11). Under the unobserved state vector, we revise the state-space model to:13where  and $\upsilon_{t} \equiv E_{t} + Z_{t} u_{t}$, $Y_{t}$ is the endogenous variable,  is the exogenous variable, and $\theta_{t}$ is the process factor that cannot be observed. Under $t = 1, \ldots ,T$ we calculate , construct specific common, national, and variable indicators. These indicators measure the extent to which each component affects $Y_{t}$. To end the state-space system, we must set a path for the unobserved state vector.

According to Cogley and Sargent [16] and Primiceri [38], to reduce the number of estimated parameters, we assume that the parameter is a random walk model as follows:

14 $$\theta_{t} = \theta_{t - 1} + \eta_{t} ,\quad \eta_{t} \sim {N}\left( {0,B} \right)$$

We limit the error covariance matrix to the block diagonal matrix $B = {blkdiag}\left( {B_{g} ,B_{c} ,B_{\upsilon } } \right)$. It is assumed that the error vectors $E_{t}$, $u_{t}$, and $\eta_{t}$ are independent of each other. Canova and Ciccarelli [10] thought that there is no correlation between $E_{t}$ and $u_{t}$. The unconditional distribution of $\upsilon_{t}$ is a $t$ multivariate distribution. This property is fundamental, indicating the flat thick tail distribution of this error.

On the other hand, there is a problem that Cogley and Sargent [16] assume a simple set for the resampling of residual conditional heterogeneity. This practice is only relevant when the coefficient decomposition is used. Therefore, we use the Bayesian method for estimation because the posterior distribution is not standard, so we further use the MCMC (Markov Chain Monte Carlo simulation) simulation to obtain the posterior distribution.

In the model estimation process, we use the Gibbs sampling algorithm to find posterior distribution using the methods of Canova et al. [12], Canova and Ciccarelli [10], and Canova and Ciccarelli [11]. Based on this, we can compute the correlation between the indicator variables, then analyze the impulse response between variables and the related empirical analysis.

The method designed in this paper allows the TVP-panel VAR. model to be transformed into TVP-PVECM if the long-term cointegration relationship is detected. To conduct an empirical analysis, we set the relationship between gold and exchange rate as:$\Delta LG_{it}$ is the logarithmic return of gold for a country's domestic currency (that is $\hat{G}_{it}$ in Eq. (7)). $\Delta LE_{it}$ is the change rate of exchange rate (that is $\hat{E}_{it}$ in Eq. (7)). If the value of $f^{\prime}$ > 0 in Eq. (7), the gold return can evade the loss risk of a country's currency purchasing power. The positive $\Delta LE_{it}$ represents the depreciation rate of a country's currency; in other words, the increase of gold price in the domestic currency. When a country's monetary authority practices an untimely policy of intervention, there may be incomplete information and additional transaction costs in the market overtimes. Therefore, the effect of demand and supply elasticity would change over time, and the impulse effect of hedging would be different. We then establish a short-term hedging equation of gold on the exchange rate with an error correction mechanism as follows:

15 $$\Delta LG_{it} = \alpha_{0it} + \sum\nolimits_{j = 1}^{p} {\alpha_{1ijt} \Delta LE_{it - j} + } \sum\nolimits_{j = 1}^{q} {\alpha_{2ijt} \Delta LG_{it - j} + } \eta_{it} (LG_{it} - a_{i} - b_{i} LE_{it} ) + \varepsilon_{it}$$

where $ecm_{it} = LG_{it} - a_{i} - b_{i} LE_{it}$ is the error correction term composed of the long-term equation, and $b_{i}$ is the hedging factor. During the estimation process of the TVP-PVECM, the short-term coefficients ($\sum\nolimits_{j = 1}^{q} {\alpha_{ijt} }$) are nonlinear dynamics.

Based on the above inferences, we establish the following hypotheses to test: (1) when $b_{i}$ > 0 means that gold can hedge exchange rate depreciation, 1 > $b_{i}$ > 0 is partial hedging, $b_{i}$ $\ge$ 1 is complete hedging. (2) when $\sum\nolimits_{j = 1}^{q} {\alpha_{1ijt} }$ > 0 (that is lower triangular matrix $A_{t}$ for setting $a_{it,}$), which means that gold returns can hedge the risk of exchange rate depreciation. (3) the impulse response of $\Delta LG_{it}$ from exchange rate risk $(\varepsilon_{\Delta LE} )$ is positive, gold is safe haven.

## Results and discussion

This study uses 15 countries as the sample, including major currency countries, major gold demand countries, and major gold-producing countries. The variables are the gold price index in each country's currency per ounce, and the exchange rate is domestic currency/SDR. The benefit of using SDR is that the impact of the U.S. can be estimated independently. A total of 720 observations are collected from the first quarter of 2009 to the fourth quarter of 2020 for the study period. The gold price and exchange rate data are from the AREMOS database. The country and code of variables are shown in Table 1.

**Table 1:** Name and Description of Variables

Sample countries/Region (code)	Australian dollar (AUD)	Canadian dollar (CAD)	Chinese yuan (CNY)	Euro (EUR.)	Indian rupee (INR)	Japanese yen (JPY)	Korean won (KRW)	Russian ruble (RUB)	Saudi Arabian riyal (SAR)	South African rand (ZAR)	Swiss franc (CHF)	Thai baht (THB.)	UAE dirham (AED)	U.K. pound (GBP)	U.S. dollar (USD)
Gold price index indexed to 01/01/1999 (National Currency Unit per troy ounce)	LG_AUD	LG_CAD	LG_CNY	LG_EUR	LG_INR	LG_JPY	LG_KRW	LG_RUB	LG_SAR	LG_ZAR	LG_CHF	LG_THB	LG_AED	LG_GBP	LG_USD
Currency units per SDR	LE_AUD	LE_CAD	LE_CNY	LE_EUR	LE_INR	LE_JPY	LE_KRW	LE_RUB	LE_SAR	LE_ZAR	LE_CHF	LE_THB	LE_AED	LE_GBP	LE_USD

Data source is AREMOS. Variables are in logarithmic form for empirical analysis

**Fig. 1:**
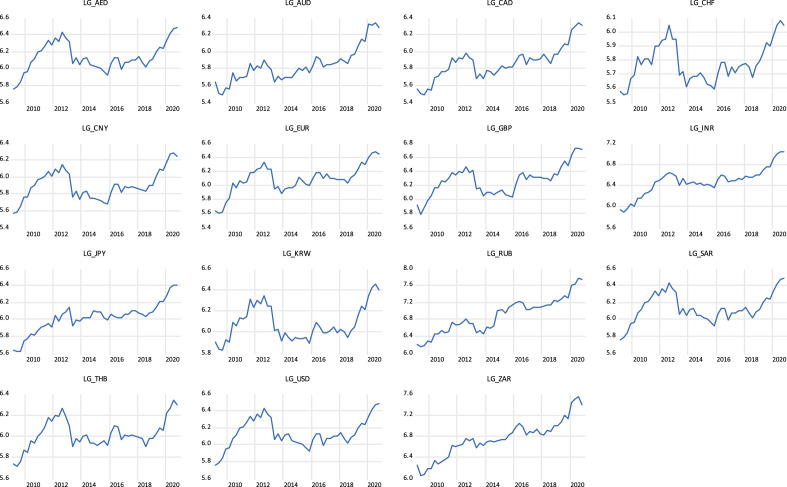
The trends of gold prices

**Fig. 2:**
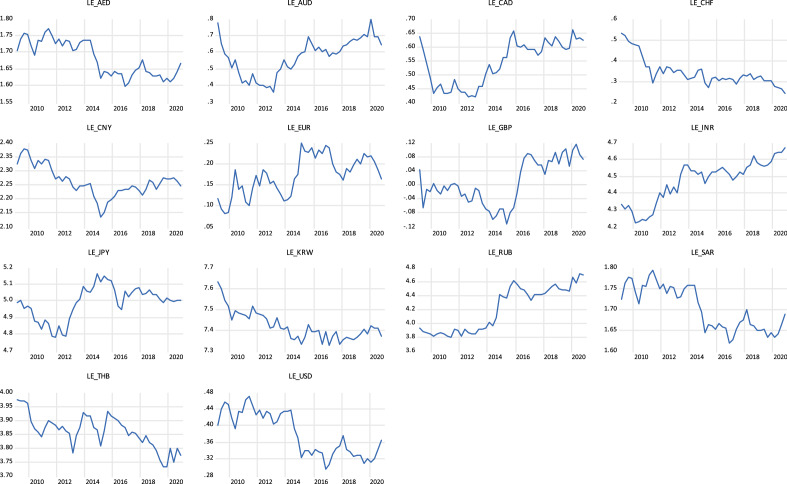
The trends of the exchange rate

Examining the trend of gold prices in countries, as depicted graphically in Fig. 1, the movement of the 15 countries are almost the same, showing an upward trend from 2009 to 2012, starting to fall from 2012 to 2014, and rebounding again after 2016, then remaining relatively stable rise from 2016 to 2020. Figure 2 shows the exchange rate trend of the 15 countries. We can see that the exchange rates are more volatile than the gold price indices. It is worth noting that all countries showed a trend of large fluctuations between 2009 and 2020. It can be seen from Fig. 2 that exchange rate fluctuations present considerable risks, while gold prices are on the rise in Fig. 1. This is why we want to examine whether gold is a safe haven for exchange rates.

Using unstable variables for the empirical model would lead to spurious results. Therefore, the stationarity of variables is necessary to be verified with the panel unit-root tests. Table 2 reports the results of panel unit-root tests, including t* test of Levin et al. [28] (LLC), t test of Breitung [9], W test of Im et al. [21] (IPS), ADF—Fisher Chi-square test of Maddala and Wu [30], and Chi-square test of PP – Fisher. AIC is used for selecting the optimal lag of the model. The results show that all variables of gold prices and the exchange rates have stationary characteristics after the first-order difference. Therefore, we use the series in first differences for estimating the model.

**Table 2:** Results of panel unit root tests

Level	LG	LE
*Method*		
Levin, Lin & Chu t* test	−3.809*** (0.001)	1.767 (0.961)
Breitung *t*-stat test	0.662 (0.746)	−0.667 (0.252)
Im, Pesaran and Shin *W*-stat test	−0.180 (0.428)	−0.761 (0.223)
ADF—Fisher Chi-square test	20.52 (0.902)	24.75 (0.736)
PP—Fisher Chi-square test	20.81 (0.893)	22.64 (0.829)
Difference	$\Delta$*LG*	$\Delta$*LE*
Levin, Lin & Chu t* test	−116.7*** (0.000)	−95.93*** (0.000)
Breitung *t*-stat test	−30.90*** (0.000)	−15.57*** (0.000)
Im, Pesaran and Shin *W*-stat test	−84.34*** (0.000)	–86.47*** (0.000)
ADF—Fisher Chi-square test	3950.8*** (0.000)	3950.8*** (0.000)
PP—Fisher Chi-square test	3950.8*** (0.000)	3950.8*** (0.000)

Done on EViews 12

We specified lags at four by minimum AIC Exogenous variables: individual effects, individual linear trends. The notation "***" implies the statistical significance at 1% level. Fisher tests are computed using an asymptotic Chi-square distribution. Values in the parentheses (.) are the p value. All other tests assume asymptotic normality. When carrying out the test as well as the estimation, all variables are formed in natural logarithm

To get a preliminary understanding of the basic statistics of data used for the model, Table 3 reports the results of the basic statistical analysis on the variables in both level and first difference terms. As the standard deviations, the risk of the level-terms is higher than the difference terms, and the risk of the gold price is higher than that of the exchange rate. The skewness coefficient shows that $\Delta$LG is left-skewed, presenting many small gains and a few extreme losses. The rest variables are right-skewed, implying many small losses and extreme gains. The kurtosis coefficients show the low peak level while the peak of difference terms is high and narrow, which means the investor should experience occasional excessive returns. The results Jarque–Bera test show that all variables are abnormal distribution.

**Table 3:** Summary statistics

	LG	LE	$\Delta$LG	$\Delta$LE
Mean	6.131	1.682	0.00044	−2.70E−05
Std. Dev	0.165	0.052	0.012	0.003
Skewness	0.109	0.087	−0.194	−0.039
Kurtosis	2.991	1.515	7.807	7.592
Jarque–Bera statistics	55.11	2558.8	26,626.1	24,144.6
Probability	(0.000)	(0.000)	(0.000)	(0.000)

Done on EViews 12

$\Delta$ represents for variables in terms of first-order difference

**Fig. 3:**
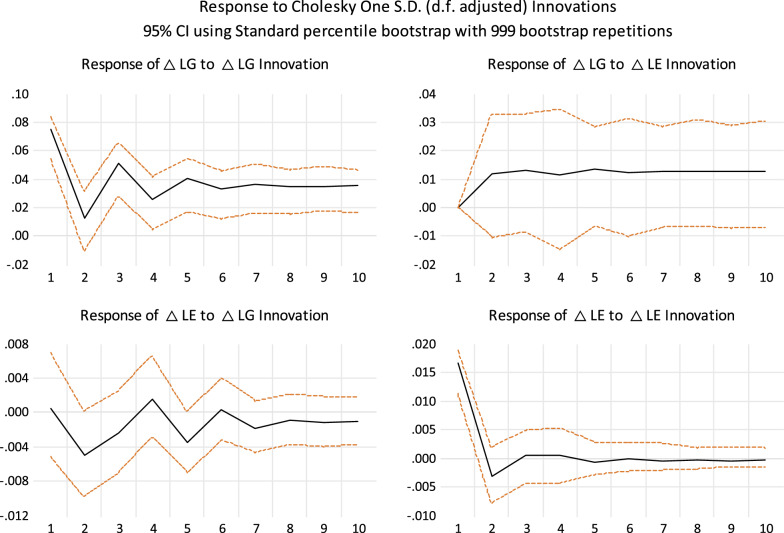
The impulse response analysis of PVECM

After determining that all variables are stationary at the first difference, we perform a cointegration test. Table 4 reports the results of panel cointegration tests. Except for the significant results of the Panel v-statistic, Panel ρ-statistic, and Group ρ-statistic, the rest shows cointegration relations between the two variables, indicating the long-term stable relationship between the gold price and the exchange rate. According to the estimation, the long-term stability $LG_{it} = \mathop {0.016}\limits_{(0.000)} + \mathop {0.736}\limits_{(0.000)} LE_{it}$.^1^ The coefficient value of 0.736 implies that gold can partially hedge against a country's currency depreciation. The error correction term derived from the long-term relationship is $ecm_{it} = LG_{it} - 0.016 - 0.736LE_{it}$. Under the long-term relationship, we use the AIC criterion to select the model with the lag period of 1 and estimate the linear panel VECM for impulse response analysis. As shown in Fig. 3, the response of $\Delta$ LE to $\Delta$ LG innovation and the response of $\Delta$ LG to $\Delta$ LE innovation are not significant. These results present gold cannot hedge exchange rate risk within a linear framework, or gold is not the safe haven for exchange rates in linear form.

**Table 4:** Panel cointegration test

Method			Test statistics	P value
Panel v-statistic			0.607	0.271
Panel ρ-statistic			− 1.755	0.039
Panel PP-statistic			− 2.869	0.002
Panel ADF-statistic			− 2.643	0.004
Group ρ-statistic			1.353	0.912
Group PP-statistic			− 2.313	0.010
Group ADF-statistic			− 1.999	0.022
Kao cointegration test			− 2.240	0.012
Johansen Fisher Panel	Hypothesized	None	19.11	0.937
Cointegration Test trace Statistic	No. of CE(s)	At most 1	55.89	0.002
Johansen Fisher Panel	Hypothesized	None	11.12	0.999
Cointegration Test max-eigenvalue Statistic	No. of C.E. (s)	At most 1	55.89	0.002

Done on EViews 12

The Pedroni's [36] statistics are asymptotically distributed as normal. The variance ratio test is right-sided, while the others are left-sided. In Johansen Fisher Panel Cointegration test, the trace and max-eigenvalue test are according to the p value of MacKinnon et al. [29]. Lags = 4

To test whether gold can avoid the dynamic risk of the exchange rate, we further estimate the TVP-PVECM. The period lag of the TVP-PVECM model selected by the AIC criterion is 1. $\sum \beta$ simultaneously is simplified to a diagonal matrix. The estimated results of the TVP-PVECM parameters are reported in Table 5, which presents posterior means, standard deviation (SD.), 95% confidence interval, CD, and Inefficiency factor. CD is a convergent diagonal statistic proposed by Geweke [20] with convergence null hypothesis, which is intended to measure the validity of the estimated coefficient, its formula is:

10 $$CD = \frac{{\overline{x}_{0} - \overline{x}_{1} }}{{\sqrt {\frac{{\hat{\sigma }_{0}^{2} }}{{n_{0} }} + \frac{{\hat{\sigma }_{1}^{2} }}{{n_{1} }}} }},\quad \overline{x}_{j} = \frac{1}{{n_{j} }}\sum\limits_{{i = m_{j} }}^{{m_{j} + n_{j} - 1}} {x^{\left( i \right)} }$$

where $x^{\left( i \right)}$ is the ith observation of a sample, ${{{(}\hat{\sigma }_{j}^{2} } \mathord{\left/ {\vphantom {{{(}\hat{\sigma }_{j}^{2} } {n_{j} }}} \right. \kern-\nulldelimiterspace} {n_{j} }}{)}^{{1/2}}$ is the standard deviation of $\overline{x}_{j}$, j = 0 or 1. When the MCMC sampling procedure is stable, the standard deviation will converge to the standard normal distribution. The smaller the value of CD and Inefficiency, the more robust the convergence and the more effective the MCMC estimation. According to the CD statistics, all parameters at the 5% significance level cannot reject the null hypothesis that samples appear to converge on the posterior distribution. These TVP-PVECM results show that the posterior estimation mean of all parameters is within the confidence interval, indicating the validity of MCMC for the model estimation.

**Table 5:** The results of TVP-PVAR model

Parameter	Mean	SD	95% Interval	CD	Inefficiency
${(}\sum \beta {)}_{{1}}$	0.0023	0.0003	[0.0018, 0.0029]	0.366	5.20
${(}\sum \beta {)}_{{2}}$	0.0023	0.0003	[0.0018, 0.0029]	0.113	3.70
${(}\sum a {)}_{{1}}$	0.0056	0.0017	[0.0034, 0.0099]	0.796	15.53
${(}\sum a {)}_{{2}}$	0.0048	0.0024	[0.0031, 0.0079]	0.787	22.10
${(}\sum h {)}_{{1}}$	0.0054	0.0015	[0.0034, 0.0091]	0.516	13.45
${(}\sum h {)}_{{2}}$	0.0056	0.0016	[0.0034, 0.0097]	0.846	10.42

Done on EViews 12

In this study, we assume as a diagonal matrix for simplicity. Compared to the non-diagonal assumption, previous experiences of Primiceri [38] and Nakajima [34] indicate that this assumption is not sensitive to the results. The following priors are assumed for the *i* -th diagonals of the covariance matrices: ${(}\sum \beta {)}_{i}^{{ - 2}} \sim Gamma \, (40,0.02)$ ${(}\sum a {)}_{i}^{{ - 2}} \sim Gamma \, (40,0.02)$ ${(}\sum h {)}_{i}^{{ - 2}} \sim Gamma \, (40,0.02)$

For the initial state of the time-varying parameter, rather flat priors are set as $\mu_{{\beta_{0} }} = \mu_{{a_{0} }} = \mu_{{h_{0} }} = 0$, and $\sum {\beta_{0} } = \sum {a_{0} } = \sum {h_{0} } = 10 \times I$. To compute the posterior estimates, we draw *M* = 10,000 observations after the initial 1000 observations are discarded. The estimates of $\sum \beta$ and $\sum \alpha$ are multiplied by 100. SD = standard deviation; CD = convergence diagnostics statistics

**Fig. 4:**
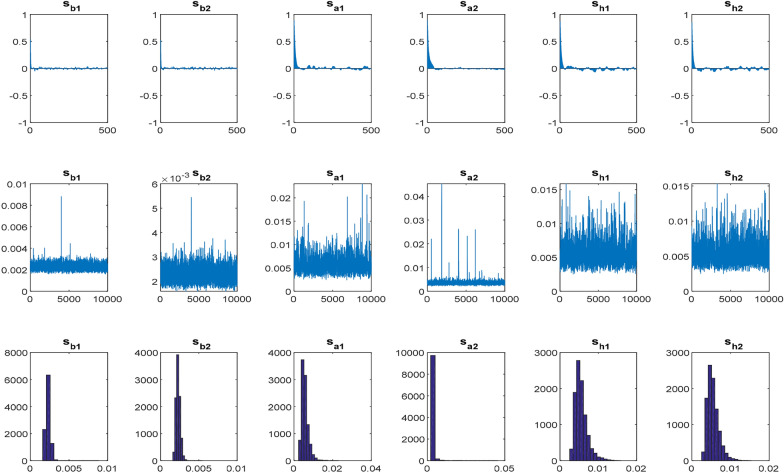
The autocorrelation parameters, the path sampling simulations, and the posterior probability distribution

Figure 4 plots the autocorrelation, risk path of sampling simulation, and posterior distribution probability density for six parameters to present the dynamic relationship between variables and related distribution paths. According to the autocorrelation graph of the parameters, AR autocorrelation in the six series and ${(}\sum h {)}_{{1}}$,${(}\sum h {)}_{{2}}$ parameters may have a process of autocorrelation and moving average ARMA. The path sampling simulation of six parameters shows that the volatility of ${(}\sum \beta {)}_{{2}}$ is large, and the probability density of the posterior distributions is skewed to the right with a high narrow peak.

**Fig. 5:**
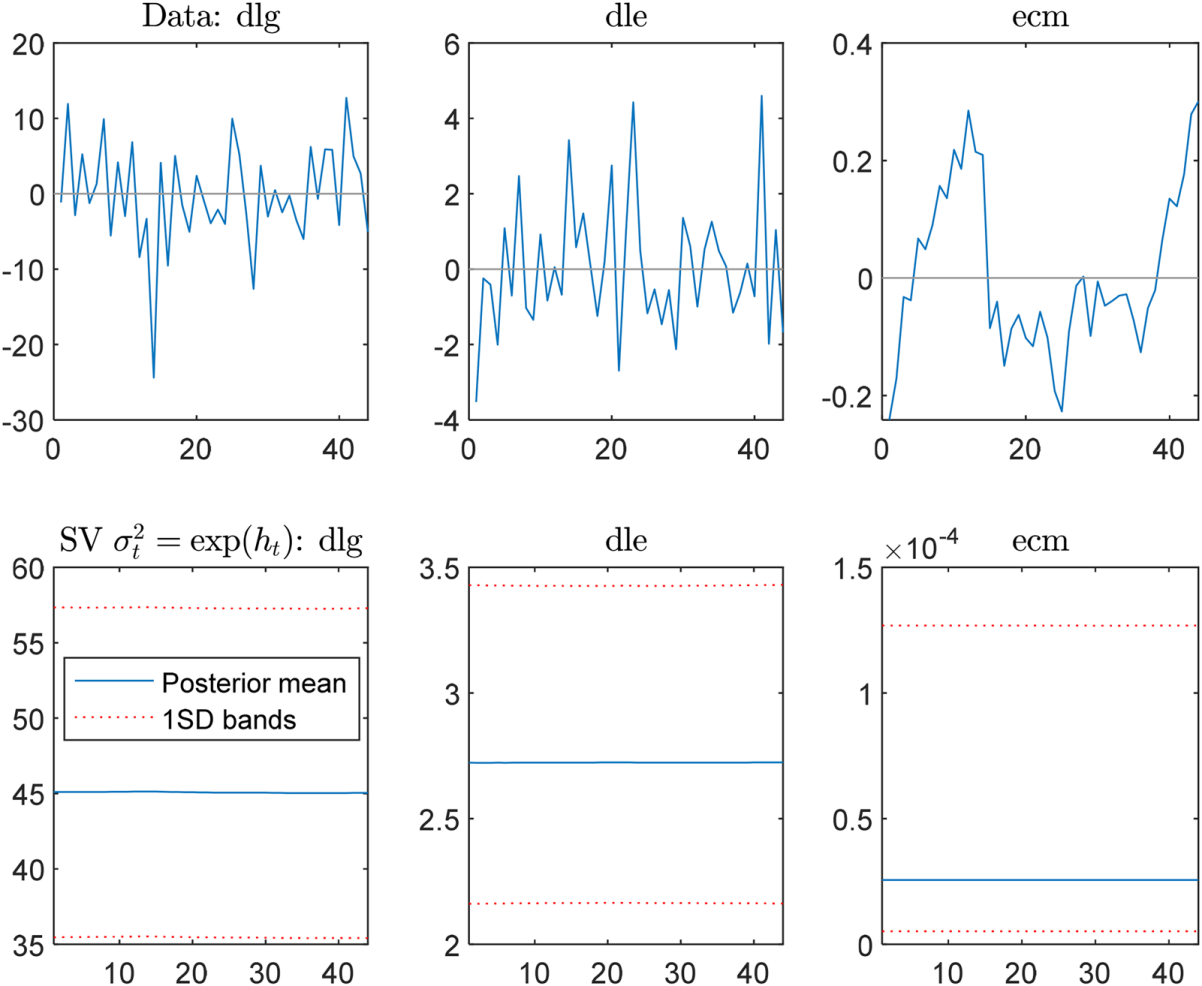
The simulated random disturbance variance of variable rate of change and average of posterior parameters

Observing the variables to simulate the fluctuation trends of variables randomly, Fig. 5 shows the simulated random fluctuations (interference variance) and the $\pm$ 1 standard deviation trend of the gold return, exchange rate volatility, and the mean of error correction terms. Structural changes induce significant volatility or risks. The random interference variation rates of gold returns are the highest compared to those of exchange rate volatilities and error corrections.

**Fig. 6:**
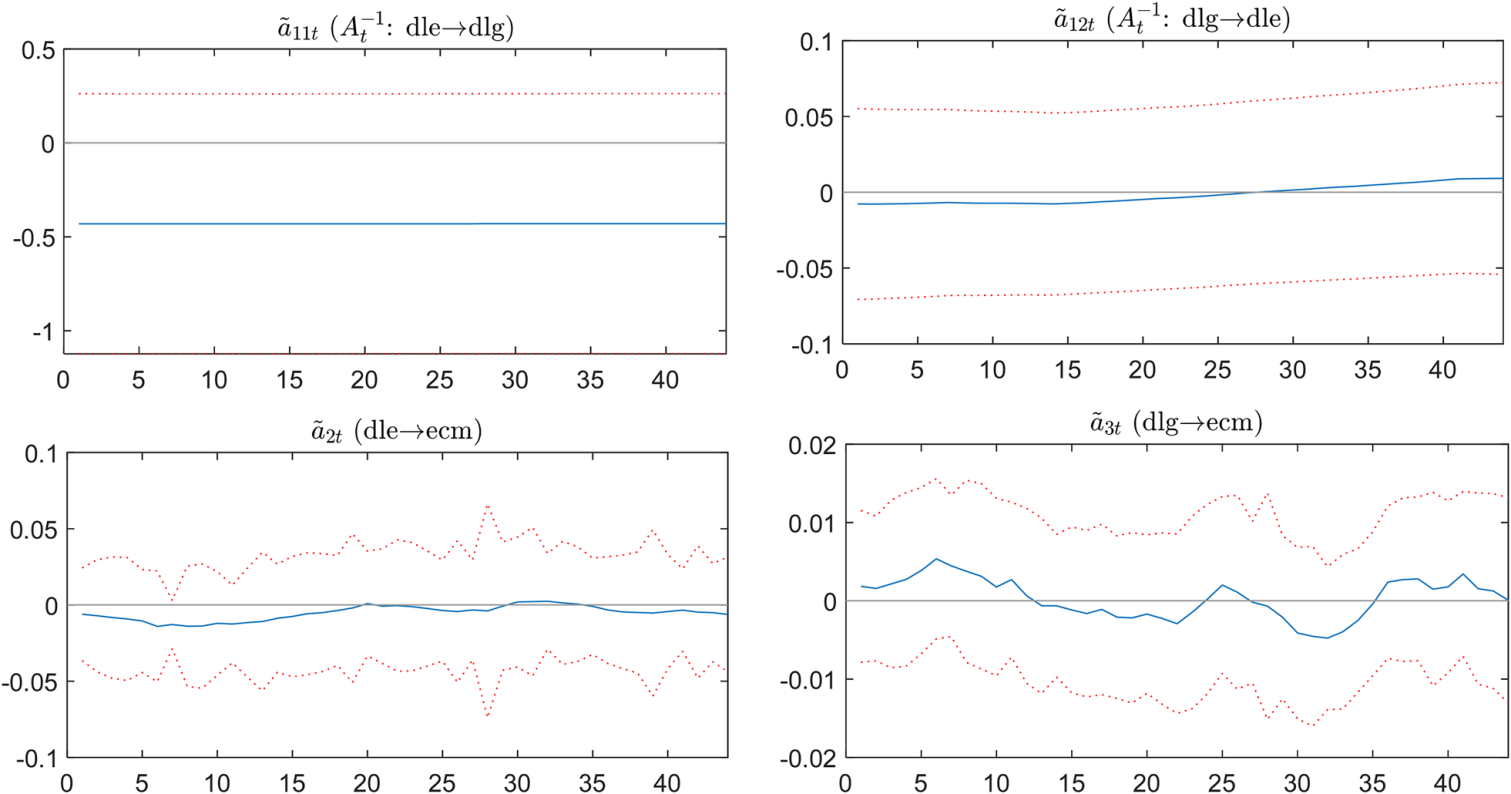
Synchronization correlation among variables

The synchronization correlation trend ($\tilde{a}_{it,}$) between variables is provided in Fig. 6, which use the lower triangular matrix $A_{t}$ for setting ($a_{it,}$*, i* = 1, 6), and have the inverse matrix of posterior distribution estimation $A_{t}^{ - 1}$. This represents the magnitude of the synchronization effect that other variables reflect for a unit structural shock in a recursive identification of one variable. The short-term dynamic effect (risk hedge) between the variables is estimated with the TVP-PVECM model.

Figure 6 shows the effect of the dynamic coefficient $\tilde{a}_{{{11}t}} (dle \to dlg)$ of the exchange rate volatilities on gold returns is negative and fixed, indicating that gold returns cannot hedge against the exchange rate risk. In other words, Short-term dynamic hedging is invalid. The dynamic coefficient $\tilde{a}_{{{12}t}} (dlg \to dle)$ is the effect of gold returns on exchange rate volatilities. The value of the dynamic coefficient is around 0, indicating that gold is having an endogenous impact. Besides, we analyze the effect of exchange rate fluctuations on error correction. It can see from $\tilde{a}_{{{2}t}} (dle \to ecm)$ that short-term imbalance can be adjusted by the exchange rate, and the error correction mechanism is within 0 adjustments. The same from $\tilde{a}_{{{3}t}} (dlg \to ecm)$, the short-term imbalance can be adjusted between 0 through the adjustment of the gold price through the error correction mechanism.

To explore how variables respond to a sudden strong and unreflective urge and how they present their behaviors to different lengths of time and different events, we perform the impulse response analysis used to determine if gold is safe haven. The impulse response analysis is an essential tool in tracking the impact of any variable on others of the TVP-PVECM model. The parameters of the traditional PVECM model do not vary with time, while the TVP-PVECM model can test for the dynamic effects between variables in another dimension. The TVP-PVECM model uses the estimated time-varying parameters to compute the impulse response at all time points. For the method to compare the impulse response magnitude of variables, considering the process of time-varying, we first fix an original impact size equal to the average of the random volatilities of the time series during the sample period, then use the synchronization correlation at each time point. To estimate the innovation process of deferred variable regression, we use the current to future period to calculate the time variation coefficient. During the final sample period, we set the coefficient to fix for simplicity. When the time level and time point are selected, a 3-dimensional impulse response pattern is generated.

**Fig. 7:**
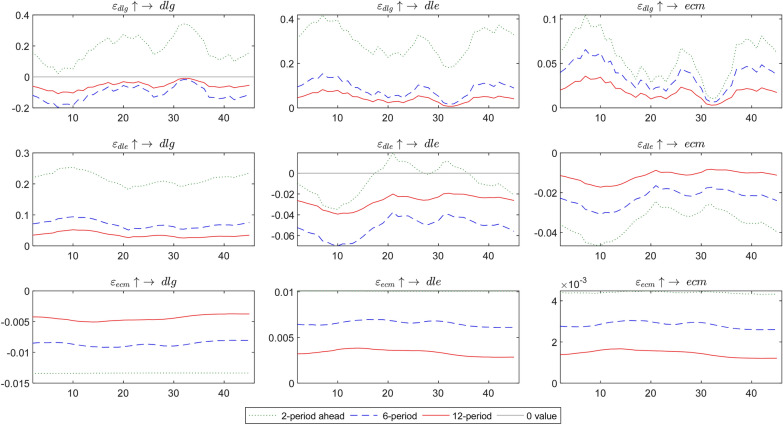
Impulse response among variables in the next 2, 6, and 12 periods

Figure 7 offers evidence of the interference between variables through the dynamic impulse response in the next 2 periods (green line), 6 periods (blue line), and 12 periods (red line). This figure focuses on comparing the effects of the short-term and long-term impulse response or the effects of risk spillover. First, the impact effect of gold price risk on exchange rate fluctuation ($\varepsilon_{dlg} \uparrow \to dle$) is positive. It changes over time with 2 periods > 6 periods > 12 periods, which means that the short-term effect is greater than the long-term effect.

The impact of gold price risk on the error correction term ($\varepsilon_{dlg} \uparrow \to ecm$) is that the short-term effect is greater than the long-term effect. The impact of exchange rate risk on gold returns ($\varepsilon_{dle} \uparrow \to dlg$) is positive, indicating that gold is a safe haven for exchange rate risk, and the short-term effect is greater than the long-term effect. The impact of gold price risk on the error correction term ($\varepsilon_{dlg} \uparrow \to ecm$) is also that the short-term effect is greater than the long-term effect. How does the adjustment of short-term imbalance affect gold returns and exchange rate fluctuations? The results of $\varepsilon_{ecm} \uparrow \to dlg$ and $\varepsilon_{ecm} \uparrow \to dle$ show that the short-term impact is more significant than the long-term impact.

**Fig. 8:**
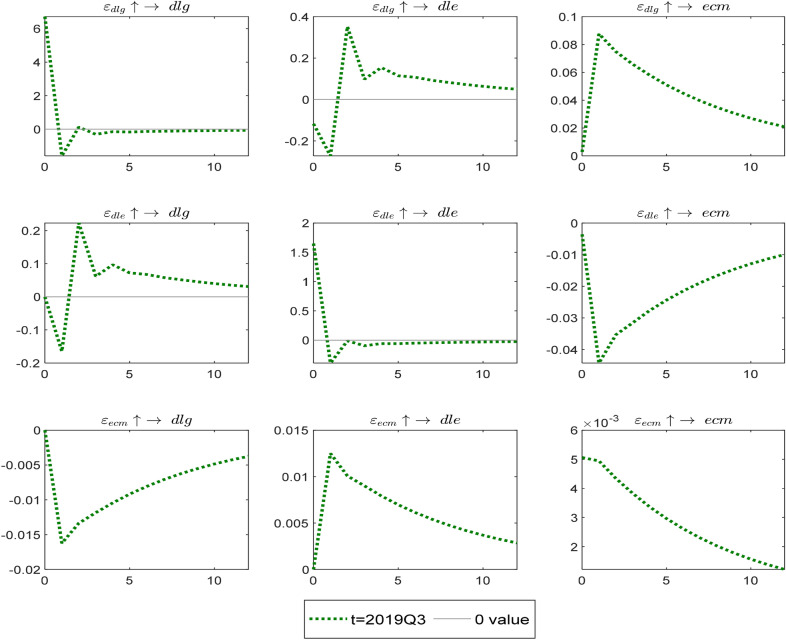
The impulse response among variables when experiencing the shock of Covid-19

Finally, we analyze the impact of the Covid-19 pandemic event. The Covid-19 pandemic occurred around the third quarter of 2019. The effect illustrated in Fig. 8 shows that the impact of the risk generated by the gold market on the foreign exchange market is negative, then becomes positive and slowly diminishes. The impact of $\varepsilon_{dlg} \uparrow \to ecm$ presents that the risk caused by the gold market first increases and then gradually decreases. From the impact of $\varepsilon_{dle} \uparrow \to dlg$, it can find that the effect is negative. Therefore, gold is not a safe haven for exchange rate risk at the beginning of the Covid-19 shock; however, the shock effect turns positive, and gold is a safe haven for exchange rate risk after the second quarter, the effect then slowly diminishes over time. The effect of $\varepsilon_{dle} \uparrow \to ecm$ implies that the risk generated by the foreign exchange market first reduces the magnitude of the imbalance correction and then slowly rises. Finally, it can see from the impact effects of the error correction $\varepsilon_{ecm} \uparrow \to dlg$ and $\varepsilon_{ecm} \uparrow \to dle$ that the magnitude of the imbalance correction first increases and then gradually decreases.

This paper constructs the TVP-PVECM for testing the hypothesis of dynamic hedging characteristics of gold on exchange rate. As the existing literature has never considered that the foreign exchange risk hedged by gold is dynamic, this article can fill the research gap in this area. The empirical results show that (1) gold can partly hedge against the depreciation of a country's currency in the long run; (2) in the short run, gold cannot hedge against the exchange rate risk when considering dynamic hedging effects; (3) the short-term impulse response effect is greater than long-term effect; (4) when facing the unexpected shocks, the gold returns have reversible reactions compared to exchange rate fluctuations; therefore, gold can regard as a safe haven for foreign exchange markets; (5) the government and investors should always be concerned about these dynamic risks and formulate effective hedging strategies to control the currency uncertainty.

Prior literature such as Wang and Lee [52], Wang et al. [55], Wang [51], and Wang and Lee [53] did not consider the correlation or risk factor that may change with time and states. How to propose relative risk dispersion and policy timely in a changing relationship is the focus of this study. For this purpose, this paper develops the TVP-PVECM model whose parameters change with time to design a transnational empirical model to test whether gold can hedge against the devaluation of a country's currency in the long run. It also applies the impulse response analysis to explore whether gold can evade the depreciation risk of a country's currency in the short run and whether gold is a safe haven for exchange rate risk when unexpected shocks occur.

## Conclusions

Managing relative risk dispersion and policy timely in a dynamic relationship is the main contribution of this study. The TVP-PVECM model is proposed to help clarify whether gold can hedge against the devaluation of a country's currency in the long run. Besides, the impulse response analysis is applied to examine if gold can stay away from the depreciation risk of a country's currency in the short run and if gold is a safe haven for exchange rate risk when an unexpected or unpredictable event occurs. Although the TVP-PVECM model does not classify the market conditions as previous literature, the impulse response analysis shows that when facing unexpected shocks, the gold returns have reversible reactions compared to exchange rate fluctuations. Therefore, gold can regard as a safe haven for foreign exchange markets. Also, we analyze the impact of the Covid-19 pandemic event and find gold is a safe haven for exchange rate risk after the second quarter; the effect then slowly diminishes over time.

Through the empirical analysis of the integration and the relationship between variables, the globalization factors of transnational dynamic time and state risks are focused, which help investors and the government deal with the reliable mechanism of the golden hedge on exchange rate risk and challenge the unexpected event. The empirical evidence of this paper provides investors and policymakers some suggestions that long-term holding of gold can partly avoid the depreciation of currencies. The dynamic risk hedging effect facing the greater risk is better than the fixed one in the short term. Also, suffering the impact of unexpected events, gold can be used as a safe haven for foreign exchange risk. Therefore, adding gold to a portfolio can help investors and legal entities reduce the volatility of returns and avoid exchange rate fluctuations. We moreover expect that the evidence provides the reasons for governments to purchase gold as an official reserve asset. In future research, the major currency countries, major gold demand countries, and major gold production countries will be classified and compared.

## Data Availability

The datasets used and/or analyzed during the current study available from the corresponding author on reasonable request.

## Footnotes

1 The parentheses (.) are the p-value.
